# Strategies to Prevent Early and Late-Onset Group B *Streptococcal* Infection via Interventions in Pregnancy

**DOI:** 10.3390/pathogens12020229

**Published:** 2023-02-01

**Authors:** Mahin Delara, Nirma Khatri Vadlamudi, Manish Sadarangani

**Affiliations:** 1 Vaccine Evaluation Center, BC Children’s Hospital Research Institute, Vancouver, BC V5Z 4H4, Canada; 2 Department of Pediatrics, University of British Columbia, Vancouver, BC V6H 0B3, Canada

**Keywords:** pregnancy, group B *Streptococcus*, infection, prenatal immunization, early-onset GBS disease, late-onset GBS disease

## Abstract

Group B *Streptococcus* is a Gram-positive bacterium that typically colonizes 10–30% of pregnant women, causing chorioamnionitis, preterm birth, and stillbirth, as well as neonatal sepsis and meningitis with early-onset disease (EOD) or late-onset disease (LOD) due to ascending infection or transmission during delivery. While there are some differences between EOD and LOD in terms of route of transmission, risk factors, and serotypes, the only preventive approach currently is maternal intrapartum antibiotic prophylaxis (IAP) which will not be able to fully address the burden of the disease since this has no impact on LOD. Probiotics and immunization in pregnancy may be more effective than IAP for both EOD and LOD. There is mixed evidence of probiotic effects on the prevention of GBS colonization, and the data from completed and ongoing clinical trials investigating different GBS vaccines are promising. Current vaccine candidates target bacterial proteins or the polysaccharide capsule and include trivalent, tetravalent, and hexavalent protein–polysaccharide conjugate vaccines. Some challenges in developing novel GBS vaccines include the lack of a correlate of protection, the potential for serotype switching, a need to understand interactions with other vaccines, and optimal timing of administration in pregnancy to maximize protection for both term and preterm infants.

## 1. Introduction

*Streptococcus agalactiae* (group B *Streptococcus*; GBS) is a Gram-positive encapsulated beta-hemolytic, chain-forming bacterium that asymptomatically colonizes the human gastrointestinal and genitourinary tracts, including in approximately one-third of healthy women [1]. GBS colonization in the genitourinary or gastrointestinal tract is reported in 10–30% of pregnant women [2] and is of particular concern due to the risk of ascending infection and/or transmission to the offspring during pregnancy or delivery [3]. 

Fetal infection with GBS is associated with adverse outcomes, such as preterm birth and stillbirth [1,4]. Perinatal infection with GBS can also cause maternal sepsis and postpartum endometritis in the mother, as well as sepsis, pneumonia, and meningitis in the newborn. In addition to the risk of death, these infections can also lead to long-term sequelae for the infant, causing damage to the central nervous system (CNS) and affecting lung function [4].

## 2. Incidence

Globally, more than 300,000 cases of neonatal GBS disease and 90,000 associated infant deaths are reported every year [5,6]. The newborn incidence of invasive GBS disease (iGBS) is estimated as 0.49 per 1000 live births, varying from 2 per 1000 live births in Southern Africa to 0.21 per 1000 in Southeast Asia [7]. Many of these are likely under-estimates since it is common in low- and middle-income countries for births to occur in the home or other community settings, and newborns with severe disease may succumb to the infection before attendance at a medical facility and a diagnosis is confirmed. In addition, many medical facilities do not have adequate resources and facilities to enable diagnostic confirmation. 

## 3. Early vs. Late-Onset Disease

Depending on the age of symptom presentation [1], GBS infection in neonates is classified as an early-onset disease (within the first week of life, EOD), late-onset disease (between 7 and 89 days of life; LOD), and ultra-late-onset disease (between 90 days and 6 months, ULOD) [8]. Some regard 72 h as the threshold between early- and late-onset disease rather than 7 days based on the rational that the routes of transmission for EOD would mostly cause GBS disease in the first 72 h, and after this time period, the routes of transmission for LOD play the main role [9]. 

Defining EOD as invasive GBS disease in infants aged 0–6 days after birth and LOD in infants 7–89 days after birth, 1 systematic review showed that EOD is approximately twice as common as LOD, although this varies between regions and has been reported in some Asian regions including Hong Kong, India, and Thailand as being up to 6 times more common [7]. Overall, the reported incidences of EOD and LOD were 0.32–0.71 and 0.04–0.65 per 1000 live births, respectively (Table 1) [7]. 

A less widely recognized category of GBS disease is prenatal-onset GBS disease when an infection occurs during pregnancy before the baby is born. Due to rare cases of GBS infection identified in miscarriage and stillbirths worldwide, prenatal-onset GBS disease is usually classified as EOD [10]. 

## 4. Transmission of GBS from Mother to Infant

Infants can be infected with GBS via multiple routes. In EOD, transmission is predominantly vertical from colonized mothers to infants through ruptured membranes or direct exposure to the maternal flora during vaginal deliveries [1,2]. The route of transmission in LOD is proposed to be primarily horizontal via colonized mucous membranes, infected breast milk, and equipment (Table 1) [11]. In prenatal-onset GBS disease, in utero acquisition is the proposed route of transmission. It is suggested that ascending GBS from the vagina will infect the fetus even in the presence of intact membranes [10].

Accordingly, the factors contributing to the development of prenatal-onset GBS disease, EOD, and LOD may also vary as discussed below.

## 5. Risk Factors

The main risk factor for prenatal-onset GBS disease [10], EOD [2], and LOD [12] is maternal GBS colonization during late pregnancy. Other common risk factors include prematurity, young maternal age, and black race [2,12]. Maternal fever during labor (>100.4 °F or >38 °C) and prolonged rupture of membranes (>18 h) are additional risk factors for EOD [2]. HIV exposure has also been associated with LOD (Table 1). Results of a systematic review showed that the probability of being infected with GBS in HIV-uninfected infants who were born to HIV-infected mothers was two times higher than in infants born to HIV-uninfected mothers, and the risk of LOD was approximately four times higher among HIV-exposed infants. The risk of developing EOD was similar to non-HIV-exposed newborns [13]. More studies are required to identify other potential risk factors and thus enable appropriate targeting of effective preventive strategies.

## 6. Biology of *Streptococcus agalactiae*

GBS is a Gram-positive organism, encapsulated by a polysaccharide layer rich in sialic acid, a common substance found in the human body [1]. This sialylated GBS capsular polysaccharide (CPS) thus makes it a challenge for the host immune system to distinguish bacteria from host cells, and therefore allows bacteria to survive in the body [1]. Importantly, CPS enables the organism to resist opsonophagocytosis by preventing the activation of key components of host opsonophagocytic-mediated mechanisms [14]. 

Bacteria also possess pili and other surface proteins, which mediate initial attachment and adherence to host epithelial cells [1]. Pili constitute the backbone protein (BP) forming the shaft and the two ancillary proteins (APs) that are attached to the cell wall by sortase-mediated transpeptidase reactions [15]. GBS includes four types of pilus, named pilus 1a (BP-1a), pilus 1b (BP-1b) [16], pilus 2a (BP-2a), and pilus 2b (BP-2b) [17]. Major protein adhesins are fibrinogen-binding proteins Fbs (including FbsA, FbsB, FbsC, or BsaB, the serine-rich repeat glycoproteins Srr1 and Srr2), the laminin-binding protein (Lmb), the *Streptococcal* C5a peptidase from group B (ScpB), the *Streptococcal* fibronectin-binding protein A (SfbA), and the GBS immunogenic bacterial adhesin BibA [18]. Moreover, bacteria produce beta-hemolysin, which helps evasion of red blood cells and C5a-ase, inactivating complement as a potent chemoattractant for neutrophils and therefore preventing the accumulation of neutrophils at the infection site [1].

## 7. Serotype Distribution

All types of GBS have a common cell wall carbohydrate antigen and are distinguished based on specific CPS expressed at high levels on its surface [1,15]. To date, ten GBS capsular serotypes have been identified (Ia, Ib, II-IX), but six serotypes (Ia, Ib, II-V) are responsible for 98% of GBS colonization and 99% of all EOD and LOD worldwide [3,4]. Moreover, studies have shown that serotype III is more commonly associated with LOD (73.0–80%) than EOD (42.9–47.0%), while other serotypes including Ia, Ib, and V are more frequently isolated in EOD than in LOD (Table 1) [7,19]. 

## 8. Clinical Manifestations and Outcomes 

Most infants with EOD become clinically symptomatic within the first 24 h. The most common form of EOD is bacteremia (78%), followed by meningitis (16%) and pneumonia (15%) [1,7]. Typical symptoms of EOD are related to the respiratory tract, including apnea, tachypnea, grunting respirations, and cyanosis. Fever is also a major symptom of infection in term infants. Preterm neonates are more likely to manifest hypothermia or be afebrile. Other clinical manifestations of EOD include lethargy, poor feeding, abdominal distention, pallor, jaundice, tachycardia, and hypotension [1].

LOD has a similar clinical presentation to EOD with bacteremia as the most common manifestation [1]. However, the risk of meningitis is higher in LOD (43%) than in EOD (16%), while the risk of bacteremia is higher in EOD (78%) than in LOD (53%) (Table 1) [7]. Other less common signs include osteomyelitis affecting mostly the humerus, pyogenic arthritis involving mainly hip and/or knee joints, and cellulitis-adenitis syndrome mostly affecting facial, submandibular, inguinal, scrotal, and prepatellar sites [20].

Meningitis, sepsis, bacteremia, and pneumonia are indicative of invasive GBS disease. The overall case fatality rate of invasive GBS is about 8.4% [21], varying from 7% to 12% in EOD cases and 4% to 9% in LOD cases [6] (Table 1). Additionally, there is a regional variation in case fatality rates with the highest rate reported in Africa (18.9%) and the lowest rate in low-income countries (4.7%) [7]. This almost four times higher case fatality rate could be related to true emergence of infant GBS disease, increased comorbidities such as HIV, and poor access to diagnostic and care services such as low use of antibiotics prophylaxis [7]. Approximately 20% of survivors suffer from neurodevelopmental impairment [22]. The increased risk of neurodevelopmental impairment is reported in both EOD (2.2–4.3%) and LOD (2.6–3.4%) cases in an 18-month follow-up [23]. Most of these adverse outcomes would be preventable by using appropriate strategies that will be reviewed in the following sections.

**Table 1:** Different features of early (EOD) and late (LOD) onset GBS disease in infants.

Characteristic		EOD	LOD
Time of onset after birth		0–6 days	7–89 days
Incidence rate in 1000 live births [7]		0.32–0.71	0.04–0.65
Route of transmission [1,2,11]		vertical via direct exposure to maternal flora	horizontal from colonized mucous membranes, infected breast milk, and equipment
Identified risk factors [2,12]		maternal GBS colonization	maternal GBS colonization
		prematurity	prematurity
		young maternal age	young maternal age
		black race	black race
		maternal fever during labor (>100.4 F /36 C)	HIV exposure
		prolonged rupture of membranes (>18 h)	
Common serotypes [7,19]	III	42.9–47%	73–80%
	Ia	22.8–28.6%	11.1–14.2%
	Ib	7.1–8.0%	2.2–5.3%
	V	7.1–10.6%	4.0–4.4%
Clinical syndrome [7]	Meningitis	16%	43%
	Bacteremia	78%	53%
Adverse outcomes	Case fatality rate [6]	7–12%	4–9%
	Neurodevelopmental impairment [23]	2.2–4.3%	2.6–3.4%

## 9. Current Strategies to Prevent GBS Disease in Infants

To avoid the aforementioned GBS-related adverse outcomes, the most effective approach currently available and widely used is intrapartum antibiotic prophylaxis (IAP), which can be implemented either via a routine culture-based or a risk-based prenatal screening strategy [24]. In a routine culture-based strategy, IAP is provided to women colonized with GBS in their prenatal cultures collected with a rectovaginal swab at 35th or 37th weeks of gestation as recommended by the Society of Obstetrics and Gynecology of Canada (SOGC) [25] or 36th to 37th weeks of gestation as suggested by the American College of Obstetrics and Gynecology (ACOG) [26]. In a risk-based strategy, IAP is offered to those with a history of perinatal GBS infection/disease, a previous infant with GBS disease, GBS bacteriuria during pregnancy, preterm birth, and/or presence of specific perinatal risk factors such as fever or prolonged rupture of membranes [24]. 

IAP has substantially reduced the incidence of EOD. For example, in the USA, this reduction has been reported as up to 82%, from 1.7 per 1000 live births in 1993 to 0.31 per 1000 live births [8,11]. However, IAP has not fully eliminated neonatal GBS disease [3], because it cannot prevent the risk of ascending infection during pregnancy and has no impact on horizontal transmission routes and thus LOD. Moreover, prenatal exposure to antibiotics can alter the infant’s gut microbiome, potentially leading to life-long consequences [27] including asthma [28], mood and anxiety disorders [29], autism, and attention deficit hyperactivity disorder [30]. Hence, improved preventive measures are urgently needed to fully address the burden of GBS disease for pregnant people and infants [3].

## 10. Potential Future Preventive Strategies

### 10.1. Probiotics

One approach to restrict or eliminate GBS colonization in pregnant women is by using oral probiotics; however, the reported efficacy of probiotics in GBS colonization prevention is uncertain [3]. As noted in Table 2, one quasi-experimental pilot study [31] and five randomized controlled trials (RCTs) have evaluated the influence of probiotics on GBS colonization in pregnant women when administered at 28–37 weeks of gestation in five different countries and regions, i.e., Australia, Austria, Canada, Taiwan, and US [32,33,34,35,36]. 

In an open-label study, 20 women were randomized to receive a once-daily oral probiotic (containing *Lactobacillus acidophilus*, *Bifidobacterium lactis*, and *B. longum*) or placebo at 28–32 and 36 weeks. In this trial, rates of GBS positivity were similar across probiotic (2/10, 20%) and placebo (2/10, 20%) groups [31]. Nevertheless, prenatal probiotic therapy successfully reduced GBS colonization in those with 90% therapy adherence compared to those with 68% therapy adherence. Of note, the investigators highlighted that those women who consumed yogurt were significantly more likely to be GBS-negative (*p* = 0.02). As this was a small study, the authors recommended a large controlled clinical trial with a diverse population. 

In a double-blind RCT conducted in Austria, 60 pregnant women at 32–36 weeks of gestational age with GBS colonization received a 14-day treatment course of a probiotic containing 4 strains of *Lactobacillus*. The probiotic failed to eradicate GBS in the majority of women (64% of the intervention group vs. 78% of the control group) [32]. The intake of these probiotics also failed to decrease the rate of preterm birth. 

In a prospective double-blind RCT, 110 pregnant women in Taiwan at 35–37 weeks of gestation were assigned to 2 probiotic capsules (containing *L. rhamnosus* and *L. reuteri*) daily or placebo until delivery [33]. They were tested for vaginal and rectal GBS colonization upon admission for delivery. A significant reduction in GBS colonization was observed in the probiotics arm (21/49, 42.9%) compared to the placebo group (9/50, 18%) (*p* = 0.007). 

In a study by Olsen et al., 34 women at 36 weeks of gestation were randomized to standard antenatal care and a daily oral dose of probiotics (*L. rhamnosus* and *L. fermentum/reuteri*) or placebo [34]. In this study, the investigators observed no significant difference in vaginal GBS rates between the control (3/21, 23.1%) and intervention groups (4/19, 21.1%; *p* = 0.7), but they noted a significant increase in vaginal commensals in the probiotics arm (*p* = 0.048). 

Finally, in a slightly larger Canadian RCT, 139 women at 35–37 weeks of gestation were allocated 2 capsules of probiotics (*L. rhamnosus* and *L. reuteri*) or a placebo [35]. The authors reported that vaginal/rectal GBS colonization rates did not differ significantly between the probiotics (9/57, 15.8%) and placebo groups (12/56, 21.43%; *p* = 0.48). Given a lower colonization rate in the probiotic arm, the investigators recommended a larger trial to assess the effectiveness of probiotic therapy. 

Another US-based randomized trial, with the participant, care provider, investigator, and outcomes assessor masking, evaluated once-daily probiotic dietary supplement or placebo for prevention of GBS colonization in 251 pregnant women at 35–37 weeks. The authors noted a difference of 1% in the two groups (18.5% vs. 19.7%) for GBS colonization [36]. 

While the aforementioned findings contribute to the growing body of research by reporting no safety concerns after using probiotics in the third trimester of normal pregnancies, using these products as an effective and feasible intervention to prevent GBS disease seems to be challenging. The main source of the lack of significant changes in the intervention groups could be rooted in the study designs such as low sample size, late recruitment in pregnancy, short intervention length, inadequate probiotic dosage, or even using a wrong product. Given that probiotics convey strain-specific health benefits [37,38], the significant increase in vaginal colonization with no impact on GBS colonization may be due to choosing a suboptimal probiotic strain for this intervention. Selecting a different route of administration (e.g., vagina) or combining different routes may also increase the likelihood of obtaining positive results. 

Some of these challenges may be well addressed in future clinical trials. As noted in Table 2, there are three ongoing trials (clintrials.gov) evaluating the use of probiotics on GBS colonization in a diverse population with varying gestation periods and probiotics [39,40,41]. In summary, with currently mixed evidence of probiotics’ effect on the prevention of GBS colonization, the data from some ongoing studies may help build consensus. 

### 10.2. Vaccines

Ultimately, to significantly reduce the global GBS burden of disease, the most effective and attractive strategy is likely to be immunization during pregnancy [15], an approach that has successfully been used for multiple infectious diseases, including influenza, pertussis, tetanus, and coronavirus disease 2019 (COVID-19) [21]. Like other vaccines, developing a GBS vaccine for use in pregnancy to protect the fetus and newborn requires efficient transplacental antibody transfer. Published data suggest that GBS vaccines would induce functionally active antibodies that can confer protection to the mothers and can cross the placenta to protect the offspring against GBS infection during the delivery and in the following months [15]. In 1976, Baker and Kasper provided evidence that infants infected with GBS type III strains lacked GBS-specific maternal antibodies [42]. Pregnant individuals with vaginal carriage of type III GBS who delivered healthy infants had a higher rate of IgG antibodies in their sera (22/29; 76%) than those who gave birth to infants with GBS disease (n = 7/29; 24%) [42]. 

Subsequent studies by other investigators also confirmed the protective role of transplacental antibodies against serotypes Ia and III [43,44,45]. These findings support the efficient placental transfer of maternal antibody-mediated protection against GBS disease. The results of these studies have led to the development of GBS vaccines and clinical studies in different populations. Currently, the most promising vaccine candidates are either capsular polysaccharide (CPS) including polysaccharide-based vaccines and polysaccharide conjugate vaccines, or protein-based subunit vaccines [15]. 

#### 10.2.1. Polysaccharide-Based Vaccines 

Polysaccharide-based vaccines are unconjugated purified native type Ia, II, or III PS vaccines and were developed in the 1980s [5,15]. These vaccines were safe and well tolerated in healthy non-pregnant adults. Defining immune response as >1 μg/mL PS type-specific antibody level, Baker and Kasper (1985) found that type II PS was more likely to elicit an immune response (occurring in 88%) than type Ia (40%) and type III (60%) at four weeks post-immunization [46]. 

In a separate study using serum PS type-specific antibody level > 2–3 μg/mL as a threshold, GBS type III CPS induced an immune response in 65% of pregnant women who were immunized at 26–36 weeks of gestation [47]. Maternal antibody levels persisted for at least 3 months after birth in 64% of serum samples of infants born to mothers with an initial antibody response to the vaccine [47]. It is believed that these vaccines induced T-cell-independent B-cell activation without the ability to generate B-cell memory response, and therefore they could not enhance immune response following booster doses, as with other bacterial polysaccharide vaccines [48,49,50]. Additionally, GBS-naïve women showed high variability in individual immune response [15,46,51]. These could be the reasons why these vaccines failed to reach phase II trials and they are not currently thought to be a promising strategy.

#### 10.2.2. Polysaccharide Conjugate Vaccines

Capsular polysaccharide–protein conjugate vaccines are the leading GBS vaccine candidates targeting at least one of six capsular types (Ia, Ib, II, III, IV, and V) that are designed to cover the majority of GBS disease-causing serotypes. The carrier protein for vaccines currently under evaluation is either tetanus toxoid (TT) or cross-reactive material (CRM)197 (a non-toxic mutant of diphtheria toxin-DT) isolated from *Corynebacterium diphtheriae* C7 [15]. Depending on how many serotypes are targeted, conjugate vaccines can be either monovalent or multivalent. Multivalent conjugate vaccines that have been employed in clinical trials so far include bivalent, tetravalent, and hexavalent formulations. 

#### 10.2.3. Monovalent Conjugate Vaccines

Monovalent GBS vaccines were initially tested in Canada in a phase I trial by Kasper et al. (1996) that included 100 healthy non-pregnant women between 18 and 40 years of age who received a type III CPS-tetanus toxoid (GBS III-TT) vaccine, polysaccharide vaccine, or saline. Compared with polysaccharide vaccine recipients (50%), the conjugate GBS vaccine recipients (90%) were more likely to respond immunologically (≥ 4-fold increase in geometric mean concentration (GMC)) at 8 weeks post-vaccination (Table 3). Moreover, the authors noted that the GBS III-TT group had a better immune response at 26 weeks post-vaccination compared with the polysaccharide group [52]. In addition, GBS III-TT was well tolerated by women with minimal reactogenicity as about 27% of recipients reported redness or swelling at the injection site which resolved in 72 h (Supplemental Table S1) [52]. 

To investigate the effect of these conjugate vaccines on pregnant women and their offspring, a subsequent clinical trial was conducted by Baker in 2003 (Table 3). In this trial, the investigators included 30 healthy pregnant women in the US at 37 weeks gestation or later. The vaccine was safe, well tolerated, and resulted in more than 50-fold increases in maternal CPS type III-specific IgG by four weeks post-immunization. The vaccine also elicited functionally active antibodies in newborns persisting until the age of 2 months with a GMC of 10 μg/mL, which was approximately 30% of III CPS-specific IgG antibodies found in maternal delivery serum [53]. The GBS III-TT conjugate vaccine was well tolerated by women with no report of vaccine-associated serious adverse events. An estimated 5% of the study population reported mild to moderate pain at the injection site (Supplemental Table S1) [53]. 

Additionally, 2 more monovalent GBS vaccines were also investigated in 35 healthy non-pregnant women. The CRM-V vaccine consisting of CRM197 was compared with a type V CPS-TT vaccine before and 2, 4, 8, 26, and 104 weeks after immunization (Table 3). The immune response did not differ significantly between these formulations. About 93–100% of vaccine recipients in each group had at least a 4-fold increase in type V CPS-specific IgG through 26 weeks, and in 85–93% of recipients, this increase lasted up to 2 years after immunization [54]. This finding indicates that the type V CPS vaccine has the potential to induce long-lasting immunity. No vaccine-associated serious adverse events were noted in this study population. Although soreness in the arm was more frequently reported by the -TT conjugate vaccine group (53.3%) compared with the -CRM (26.7%) or placebo (40%), these differences were not statistically significant (Supplemental Table S1).

#### 10.2.4. Multivalent Conjugate Vaccines 

**Figure 1:**
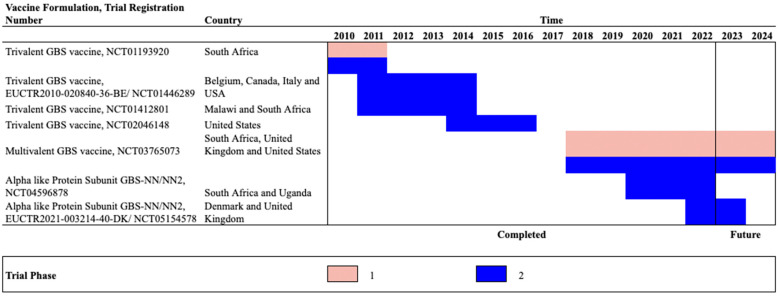
Summary of GBS vaccine products timeline. This figure describes the timeline of clinical trials in late-stage clinical development. While three phase II trials are on-going, five have been completed and data are publicly available for three studies. Among three ongoing trials, one for multivalent GBS vaccine has phase I and II combined trial; note phase I and phase II study population differs by gestation weeks. Promising results from the completed studies and pending results from on-going studies increase the likelihood of maternal immunization against GBS to prevent GBS infections among mothers and their infants.

**Figure 2:**
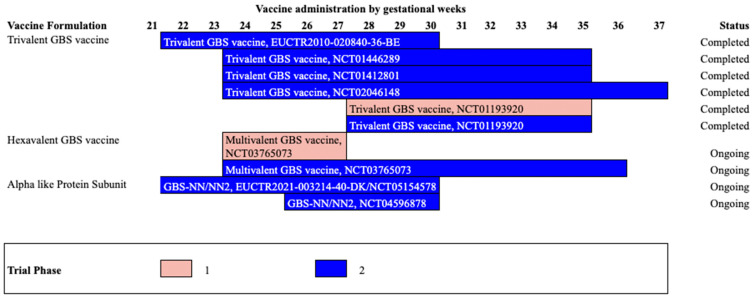
Summary of GBS vaccine target population by gestational period for vaccine administration. As of 5 August 2022, seven phase I and II trials for trivalent and multivalent conjugate and Alpha-like protein subunits vaccines against GBS in the US National Library of Medicine’s Clinical Trial database were found; of these, two phase II studies were listed in the EU Clinical Trials Register.

Monovalent conjugate vaccines were immunogenic for specific serotypes. Subsequently, multivalent conjugate vaccines were developed to obtain broader coverage against the most common disease-causing strains circulating worldwide. These vaccines have been tested in different clinical trials (Table 3) with different timelines (Figure 1) and target populations (Figure 2) described below. 

Bivalent GBS vaccines were evaluated in a phase II trial by Baker et al. (2003) that included 75 US healthy adults between 18 and 45 years of age who received a monovalent GBS II-TT conjugate, a monovalent GBS III-TT conjugate, or a bivalent GBS II-TT/III-TT conjugate vaccine (Table 3). The investigators found a ≥4-fold increase in GBS II/III CPS-specific IgG in the post-immunization serum samples among 80–90% of the bivalent vaccine group. As a result, the response was comparable to those who received GBS-II-TT (90%) or GBS-III-TT (90%) monovalent vaccines [55]. Data from this trial support the concept of multivalent vaccines including serotypes commonly causing invasive GBS disease. Furthermore, no serious side effects were observed among the study participants. 

The safety and superiority of bivalent conjugate vaccines in inducing immune response resulted in evaluating the safety and immunogenicity of a trivalent GBS vaccine based on type Ia, Ib, and III CPS conjugated to CRM197 in one phase Ib/II [56,57] and three phase II studies in pregnant women aged 18–40 years old at 24–35 weeks of gestation (Table 3) [58,59,60,61]. 

In a phase Ib/II trial conducted by Madhi et al. (2016) among 380 South African pregnant women immunized at 28–35 weeks of gestation in 2010–2011, trivalent GBS conjugate vaccines elicited functional antibodies 13–23-fold higher in the vaccine group compared with the placebo (Table 3). With no significant difference, mother-to-offspring transfer ratio of GBS-specific antibodies was observed in each group with infant antibody levels reaching 49% to 79% of maternal antibody levels at birth [57]. 

An additional study by Madhi et al. (2017) noted that infants born to those who were immunized against the trivalent GBS vaccine in pregnancy had antibody GMCs that were significantly higher compared to the placebo group at each time point including birth and on days 43 and 91 (Supplemental Table S2). Levels of antibodies against GBS in the intervention group declined over time to 41–51% and 26–35% of the levels found at birth by day 43 and day 91, respectively [56]. As noted in Supplemental Table S1, local adverse events (i.e., pain or swelling at the injection site) were similar across vaccine (35%) and placebo (30%) groups among pregnant women [57]. However, systemic events such as fatigue were higher in the vaccine group (60%) compared with the placebo (46.3%) group [57]. The investigators reported one death in the GBS vaccine group unrelated to the vaccine [57]. 

In a phase II study of a trivalent GBS vaccine that was conducted in Belgium, Canada, Italy, and the US in 2011–2013, 86 healthy pregnant women (24–35 weeks of gestation) from the 18–40 years age group were randomized to evaluate the placental transfer of anti-GBS type-specific CPS antibodies [59]. In this study, the GMC increased 16-fold, 23-fold, and 20-fold against serotypes Ia, Ib, and III by delivery. Maternal-to-infant transfer ratios of the antibodies against serotypes Ia, Ib, and III at delivery varied between 66% and 79%. 

In a study by Fabrini et al., the investigators assessed the functional activity of maternal and cord antibodies for trivalent GBS vaccines [60]. As seen in Supplemental Table S2, antibody-mediated group B *Streptococcus* opsonophagocytic bacterial killing assay (OPKA) was higher in maternal serum at delivery in the vaccine group compared to the placebo group for all three serotypes Ia, IIb, and III. Local and systemic events were higher in the vaccine group compared to the placebo group (Supplemental Table S1). Pain at the injection site was the most common local reaction reported across the trivalent GBS vaccine group (N = 35/49, 71%) compared with the placebo group (N = 12/34, 35%) [62]. Of note, systemic events and myalgia were frequently reported in the vaccine group (27/49, 55%) compared to the placebo group (3/34, 8%). 

Swamy et al. conducted a randomized placebo-controlled phase II trial in the US in 2014–2015 evaluating the safety and immunogenicity of a maternal trivalent GBS vaccine in 75 pregnant women between the ages of 18 and 40 years and their infants (Table 3). It was highlighted by the investigators that serotype-specific IgG GMCs were 13–23-fold higher in the vaccine group compared with placebo recipients on day 31 and until 90 days postpartum [61]. Moreover, the antibody transfer ratio was 62% to 82% among vaccinated groups. As expected, local and systemic events were higher in the vaccine group compared with the placebo group. Local reactions such as pain at the injection site were higher in the vaccine group (50%) compared with the placebo group (31%) (Supplemental Table S1). Similarly, systemic events such as fatigue was higher in the vaccine group (38%) compared to the placebo group (23%). Comparable serious adverse events were observed across the vaccine (15%) and placebo (16%) groups in maternal (i.e., premature separation of placenta, pre-eclampsia, premature delivery) and fetal outcomes (i.e., congenital cataract, ventricular septal defect). None were fatal or vaccine related. 

Trivalent GBS vaccines were also tested in the pregnant population with medical conditions (Table 3). In a non-randomized phase II trial on 270 pregnant women conducted in Malawi and South Africa from 2011 to 2012, Heyderman et al. evaluated GBS vaccination in a HIV-positive population [58]. They found trivalent GBS vaccines were less immunogenic in women living with HIV with both a high CD4+ count of >350 cells/µL (N = 81, mean GMCs ranged from 1.03 to 2.4 μg/mL against serotypes Ia, IIB, III) or a low CD4+ count of ≤350 cells/µL with >50 cells/µL (N= 83, ranging from 1.07 to 2.07 μg/mL) compared with those who were HIV negative (N = 83, with mean GMC ranging from 3.61 to 4.08 μg/mL) [58]. Similarly, lower GMCs were observed in infants born to HIV positive mothers (0.52–1.62 μg/mL) compared to HIV negative individuals (2.67–3.91 μg/mL). In this study, maternal-to-infant transfer ratio was assessed using infant’s mean GMC of antibodies in blood collected within 72 h of birth divided by maternal antibody GMCs in blood collected at delivery. Accordingly, the transfer ratio was found to be similar across three groups (49% to 72%). Local and safety events differed among groups based on HIV status (Supplemental Table S1). Local reactions such as pain and swelling at the injection site were lower in low (16/87, 18%) or high (26/88, 30%) CD4 cell count HIV groups compared with those in the uninfected HIV group (35/90, 39%). Similarly, systemic events such as chills, nausea, malaise, myalgia, and fever were lower in low (35/87, 40%) or high (48/88, 55%) CD4 cell count HIV groups compared with those in the uninfected HIV group (53/90, 59%). 

While the results from trials testing trivalent conjugate vaccines were promising, quadrivalent conjugate vaccines were formulated to target more common disease-causing strains circulating worldwide. These vaccines were not tested in the pregnant population but the results of a feasibility study conducted in the US including 40 healthy adults evaluating varying doses showed that the vaccine was well tolerated and produced a moderate immune response in adults with pre-existing GBS antibodies [63]. This study included individuals who were GBS antibody naïve and a separate group of individuals with pre-existing GBS antibodies. In GBS-naïve participants, seroconversion rates and 4-fold rise in antibody GMC after 6 weeks of vaccination were 33% (GMC = 5.2), 17% (GMC = 3.6), and 70% (GMC= 43.4), respectively, against serotypes Ia, II, and III, respectively (Table 3). In contrast, in those with evidence of prior GBS exposure, seroconversion rates and GMCs for type Ia and III were 90% (GMC = 73.4) and 40% (GMC = 22.2), respectively. As investigators did not find any dose-response effect, they recommended identifying better vaccine candidates. Eleven participants (27%) in the vaccine group experienced mild adverse events following immunization, six individuals (15%) reported pain and tenderness at the injection site, and six subjects (15%) reported headache, nausea, and malaise (Supplemental Table S1). 

While no study data are yet publicly available for an on-going trial on hexavalent GBS vaccine in pregnant women, data look promising from a phase 1/2 placebo-controlled dose-escalation trial in non-pregnant adults [64]. Recently, Absalon et al. evaluated a novel hexavalent GBS conjugate vaccine in 364 healthy, non-pregnant adults aged 18–49 years [64]. A 40-fold to 56-fold rise in geometric mean titres was observed in the vaccine groups ranging from 5 to 20 microgram with or without aluminum phosphate adjuvant compared with the placebo group (Table 3). No serious adverse events associated with the vaccine were reported. Pain at the injection site was frequently reported across all vaccine dose-escalation groups (ranging from 25% to 53.5%) compared with 15.4% of individuals in the placebo group (Supplemental Table S1). Systemic events such as nausea, vomiting, diarrhea, headache, fatigue, muscle pain, joint pain, and fever were similar across vaccine and placebo groups. Consequently, this hexavalent GBS vaccine was found to be safe and immunogenic in healthy adults aged 18–49 years [64]. Currently, data collection is underway for an on-going clinical trial (NCT03765073) in the pregnant population [65].

#### 10.2.5. Protein-Based Vaccines

Protein subunit vaccines are an alternative approach designed to target proteins such as N-terminal domains of the Alp-protein family. With a potential to cover Alp variants conserved across most GBS serotypes, these vaccines may have higher strain coverage than conjugate vaccines [21]. Currently, there is an ongoing interest in the use of protein-based vaccines against GBS in pregnancy. As noted in Table 3, there are two phase II studies underway to evaluate the safety and immunogenicity of the Alpha protein-like subunit GBS-NN/NN2 vaccine in mothers and infants as follows. 

The first trial will investigate 4 vaccination regimens in 270 healthy pregnant women at 21–23 weeks of gestation [66,67]. This study will evaluate the safety profile and levels of GBS IgG specific to AlpN Proteins (RibN, Alp1N, Alp2N, and AlpCN) in mothers and infants up to 6 months post-delivery. In the second trial, there are four groups: vaccine and placebo groups for HIV positive and HIV negative population. This trial will assess the immunogenicity, safety, and birth outcomes following immunization up to 6 months post-delivery among 100 pregnant women living with HIV positive and 100 pregnant women who are HIV negative, aged 18–40 years old, and are between 2 and 30 weeks of pregnancy inclusive [68]. 

**Table 3:** GBS vaccines investigated in clinical trials on pregnant or non-pregnant populations using different databases.

Vaccine Formulation	Registry Number	Country/ Region	Clinical Phase (Status)	Study Duration	Age Group, Years	Gestation Weeks	Total Study Population	Population, N (Groups)	Time Points for Immunogenicity Assessment	Immunogenicity Results: Mean GMC (95% CI)	Systemic Events	Reference
**Monovalent GBS III-TT conjugate vaccine**	N.R.	Canada	NR	NR	18–40	Non-pregnant	100	Vaccine group: III-TT58 mg: 30 III-TT 14.5 mg: 15 III-TT 3.6 mg: 15 III CPS, 50 mg: 30 Placebo group: Saline, NA: 10	8 weeks	Vaccine group: III-TT 58μg: 4.53 (1.92–10.70) III-TT 14.5μg: 2.72 (0.95–7.76) III-TT 3.6μg: 1.10 (0.40–3.02) III CPS 50μg: 1.41 (0.59–3.37) Placebo group: Saline: 0.16 (0.06–0.45)	N.R.	Kasper et al., 1996 [52]
**Monovalent GBS III-TT conjugate vaccine**	N.R.	USA	Phase 1 (Completed)	NR	18–45	>37	30	Vaccine group: 20 Placebo group: 10	4 weeks	Vaccine group: III-TT: >1.0 μg/mL Placebo group: Saline: 0.06 μg/mL	N.R.	Baker et al., 2003a [53]
**Bivalent GBS conjugate**	N.R.	USA	Phase 2 (Completed)	NR	18–45	Non-pregnant	75	Vaccine group: Monovalent GBS II–TT (3.6 mg of CPS) n = 25; Monovalent GBS III–TT (12.5 mg of CPS) n = 25; Bivalent GBS II–TT/III–TT n = 25 No placebo group	8 weeks	II-TT (3.6 mg) group: IgG: 5.7 (0.7–259; 2.9–11.3) IgM: 10.9 (1.1–61.9; 7.4–16.1) IgA: 2.1 (0.2–28.1; 1.2–3.5) III-TT (12.5 mg) group: IgG: 2.0 (0.05–402; 0.7–5.8) II-TT/III-TT (3.6/12.5 mg) group: IgG: 13.1 (0.4–571; 5.6–30.6) IgM: 10.4 (0.3–81.5; 6.8–16.1) IgA: 2.1 (0.1–153; 1.1–4.1)	No serious adverse effects in any group. Mild systemic symptoms associated with low-grade fever in 2.6% of Bivalent group	Baker et al., 2003b [55]
**Trivalent GBS conjugate vaccine**	NCT01193920	South Africa	Phase1b/2 (Completed)	2010–2011	18–40	28–35	380	Vaccine group (non-pregnant): GBS vaccine 20 μg n = 40 Placebo group: (non-pregnant): Placebo n = 20 Vaccine group (Pregnant) GBS vaccine 0.5 mg n= 80; GBS vaccine 2.5 μg n = 80; GBS vaccine 5.0 μg n= 80; Placebo Group (Pregnant) n= 80	At delivery	Vaccine group: At delivery GBS vaccine 2.5 μg Serotype Ia: 5.57 (2.25–14) Serotype Ib: 2.10 (0.65–6.78) Serotype III: 0.50 (0.10–2.46) Placebo group: At delivery GBS vaccine 2.5 μg Serotype Ia: 0.21 (0.15–0.31) Serotype Ib: 0.16 (0.07–0.37) Serotype III: 0.05 (0.03–0.07)	In non-pregnant vaccine vs. placebo groups: Chills: (37.50% vs. 30%) Fatigue: (72.50% vs. 60%) Pyrexia: (15.00% vs. 0%) In pregnant participants: vaccine 0.5mg, 2.5mg,5mg vs. placebo Chills: 20.00% vs. 8.75% vs. 10% vs. 11.25%) Fatigue: (48.75% vs. 42.5% vs. 53.75% vs. 46.25%); Pyrexia: 6.25% vs. 1.25% vs. 5% vs. 2.5%) 1/80 (1.25%) death in GBS vaccine 2·5 μg group in pregnant women	Madhi et al., 2016 [57]
**Trivalent GBS conjugate vaccine**	NCT01412801	Malawi, South Africa	Phase 2 (Completed)	2011–2012	18–40	24–35	270	GBS vaccine in: HIV-infected, low CD4 cell count >50 to ≤350 group: n = 91 HIV-infected, high CD4 cell count >350 cells per μ group: n = 89 HIV-uninfected group: 90	4 Weeks	Serotype Ia HIV-infected, low CD4 cell count group: 2.68 (1.74–4.10) HIV-infected, high CD4 cell count group: 3.26 (2.14–4.98) HIV-uninfected group: 6.63 (4.37–10) Serotype Ib HIV-infected, low CD4 cell count group: 2.62 (1.62–4.24 HIV-infected, high CD4 cell count group: 3.68 (2.38–5.70) HIV-uninfected group: 5.35 (3.63–7.87) Serotype III HIV-infected, low CD4 cell count group: 1.51 (0.97–2.35) HIV-infected, high CD4 cell count group: 1.31 (0.85–2.02) HIV-uninfected group: 5.35 (3.66–7.83)	any systemic reactions (chills/ nausea/ malaise/ myalgia/ arthralgia/ headache/ fatigue/ rash/ fever) in low CD4 vaccinated vs. high CD4 vaccinated vs. uninfected group: (40% vs. 55% vs. 59%)	Heyderman et al., 2016 [58]
**Trivalent GBS conjugate vaccine**	NCT01446289/ EUCTR2010-020840-36-BE	Belgium, Canada, Italy	Phase 2 (Completed)	2011–2013	18–40	24–35	86	Vaccine group: n = 49 Placebo group: n = 34	At delivery	Vaccine group: Serotype Ia: 5.22 (3.37–8.10) Serotype Ib: 2.41 (1.48–3.94) Serotype III: 1.90 (1.15–3.12) Placebo group: Serotype Ia: 0.37 (0.22–0.63) Serotype Ib: 0.13 (0.07–0.23) Serotype III: 0.11 (0.06–0.19)	Vaccine vs. placebo group: Chills: 4/49 (8% vs. 8%) Malaise:14/49 (28% vs. 26%) Myalgia:27/49 (55% vs. 8%) Arthralgia: 4/49 (8% vs. 26%) Headache: 16/49 (32% vs. 61%) Fatigue: 31/49 (63% vs. 76%)	Donders et al., 2016 [59]
**Trivalent GBS conjugate vaccine**	NCT02046148	USA	Phase 2 (Completed)	2014–2015	18–40	24–35	75	Vaccine group: 49 Placebo group: 26	At delivery	Vaccine group: In mothers: sIgA- Serotype Ia: 283 (104–770) Serotype Ib: 418 (151–1155) Serotype III: 112 (44–286) IgG- Serotype Ia: 0.64 (0.29–1.42) Serotype Ib: 0.10 (0.04–0.24) Serotype III: 0.41 (0.21–0.79) Placebo group: In mothers: sIgA- Serotype Ia: 2.94 (0.89–9.68) Serotype Ib: 4.17 (1.37–13) Serotype III: 0.45 (0.13–1.58) IgG- Serotype Ia: 0.12 (0.05–0.28) Serotype Ib: 0.04 (0.02–0.11) Serotype III: 0.15 (0.07–0.30)	Vaccine vs. placebo group: Fatigue: (38% vs. 23%) Nausea: (15% vs. 12%)) Headache: (13% vs. 12%)) Loss of appetite: (13% vs. 8%) Myalgia: (4% vs. 8%) Arthralgia: (4% vs. 8%) Chills: (2% vs. 4%) Rash: (2% vs. 0%) Urticaria: (2% vs. 0%)	Swamy et al., 2020 [61]
**Quadrivalent GBS**	N.R.	USA	Phase 1 (Completed)	NR	18–40	Non-pregnant	40	Vaccine group: n = 40 Absence of placebo group	6 weeks	Vaccine group: GMC of type-specific antibody (range) Ia: 5.2 (1–260) IIb: Not done II: 3.6 (0–72) III: 43.4 (2–496) Placebo group: No placebo group	Vaccine group: nausea, malaise, myalgia, or headache: 15% No placebo group	Kotloff et al,1996 [63]
**Hexavalent GBS conjugate vaccine**	NCT03170609	USA	Phase 1/2 (Completed)	2017–2018	18–49	Non-pregnant	365	Vaccine group: GBS6 5 μg with AlPO4: n = 52; GBS6 5 μg without AlPO4: n = 52; GBS6 10 μg with AlPO4: n = 52; GBS6 10 μg without AlPO4: n = 52; GBS6 20 μg with AlPO4: n = 52; GBS6 20 μg without AlPO4: n = 52; Placebo group: n = 52	2 Weeks	Vaccine group: Ia: 17.829 (7.542–42.149) Ib: 1.948 (0.785–4.836) II: 31.786 (17.490–57.770) III: 3.766 (1.887–7.517) IV: 7.018 (4.518–10.903) V: 6.760 (3.230–14.146) Placebo group: Ia: 0.755 (0.372–1.536) Ib: 0.034 (0.019–0.059) II: 0.187 (0.126–0.279) III: 0.081 (0.054–0.122) IV: 0.046 (0.027–0.078) V: 0.143 (0.084–0.244)	Vaccine vs. placebo groups: fatigue or tiredness and headache: 2% vs. 2%	Absalon et al., 2021 [64]
**Hexavalent conjugate vaccine**	NCT03765073	USA, UK, South Africa	Phase 2 (Ongoing)	2019–2024	18–40	24–36	586	N.R	N.R.	N.R.	N.R.	Pfizer [65]
**Alpha like Protein subunit**	NCT05154578/ EUCTR2021-003214-40-DK	Denmark, South Africa	Phase 2 (Ongoing)	2022–2023	18–64	22–30	270	N.R	N.R.	N.R.	N.R.	Minervax (a) [67]; Minervax (c) [66]
**Alpha like Protein subunit**	NCT04596878	South Africa, Uganda	Phase 2 (Ongoing)	2020–2022	18–40	26–30	205	N.R	N.R.	N.R.	N.R.	Minervax (b) [68]

Footnote: Clinical trial registries maintained by the WHO, US National Library of Medicine’s Clinical Trial database, EU Clinical Trials Register, Health Canada’s Clinical trial search database, Chinese Clinical Trial Registry, BMC’s ISRCTN registry, and Clinical Trial Registry-India for GBS vaccine trials in pregnant women studying prevention of GBS infections in their infants. Safety events were calculated from given proportions in references: Kasper et al., 1996, Baker et al., 2003, and Heyderman et al., 2016. GBS: group B *Streptococcus*; SAE: serious adverse events, AE: adverse events, CPS: capsular polysaccharide, HIV: human immunodeficiency virus, NR: not reported, OPKA: opsonophagocytic bacterial killing assay, ELISA: enzyme-linked immunosorbent assay, GMC: geometric mean concentration, sIgA: serotype-specific secretory immunoglobulin A, IgG: immunoglobulin G.

## 11. Challenges 

Currently, multiple studies are testing different vaccine candidates targeting either GBS capsule or proteins, but they are facing many challenges. Considering the low incidence of neonatal GBS disease, large numbers of immunized pregnant women would be required to conduct clinical phase III efficacy trials. Specific characteristics of the target population with specific follow-up requirements both for the mother in late pregnancy and the offspring in early life and the progressive nature of GBS infection before and after childbirth require adequate safety oversight and invasive procedures for blood and CSF sampling, as well as bacteriologic assessments [69]. These factors would make efficacy trials more challenging when compared with other vaccines targeted at other pathogens. While these are logistically challenging, the identification of a serologic correlate of protection that may be used as a pathway to licensure is one attractive approach [5,7]. However, in the absence of such correlate estimates as a direct evidence pre-licensure, effectiveness studies may rely on mathematical models which may be biased by imperfect assumptions. Nevertheless, post-marketing confirmatory effectiveness studies should be conducted through large multicenter phase IV studies to assess herd protection and the impact of vaccination on strain variation, carriage, and GBS disease burden in neonates.

Serological correlates of protection (CoP) as substitute endpoints that are acceptable to vaccine regulators have not been defined yet. Protective thresholds may vary according to epidemiological settings, distribution of serotypes, and host factors. However, a unique and reliable predictor of clinical benefit for the licensure of GBS vaccines should ideally be identified to facilitate vaccine licensure. Determining an antibody-based marker with a single, universally accepted threshold for licensure assessments would be ideal. For instance, IgG binding antibody in cord blood has the potential to serve as the CoP if it is highly correlated with functional antibodies and shows similar kinetics to antibodies induced after natural infection. In this respect, absolute and relative disease risk estimates as functions of infant antibody concentration can serve as CoPs if they are adjusted for confounding factors such as maternal age and gestational age. However, establishing an antibody concentration threshold as an indicator of high protection is challenging due to existing uncertainties rooted in unmeasured confounders, imperfect causal mediation, and regional and/or serotyping variations in point and confidence estimates [70].

Establishing a rigorous estimate of protective thresholds also requires consistency across clinical trials in terms of study designs, sample size, population characteristics, target antigens, methodological assays, and time point assessments. Instead of identifying a single correlate of protection, an alternative approach is establishing a composite correlate of multiple measures that may include antibody levels and/or functions, placental antibody transfer ratios, or even detection of maternal or offspring colonization [69].

The optimal immunization schedule (dose amount and number of doses) as best timing of vaccination need to be determined [71]. The appropriate timing of immunization before birth should be considered to optimize maternal antibody transfer to the offspring. Regardless of safety concerns, immunization in early pregnancy may prevent EOD but not LOD due to potential antibody waning in the first few weeks of life. However, if a vaccine is highly immunogenic in pregnancy, with high transplacental transfer ratio of maternal antibodies to the offspring and long duration of immune response, immunization in early pregnancy may have the potential to prevent LOD as well. For instance, a trial assessed type V GBS vaccines in non-pregnant people and showed that the vaccine elicited antibodies that persisted up to 2 years after vaccination [54]. Accordingly, it is possible that maternal antibodies transferred to the newborn may remain in infant’s sera for sufficient duration to prevent LOD. However, no additional trial has investigated the vaccine targeting V serotype to confirm the previous findings specifically in the pregnant population. 

A wide window period in the late 2nd or early 3rd trimesters could also be a good option for optimal protection against neonatal GBS disease, as has been identified for pertussis where, like GBS, the primary goal is protection of infant disease. As defined by the WHO, one of the preferred product characteristics for a GBS vaccine is a one dose regimen targeting pregnant women in the 2nd and 3rd trimesters [21]. Preconception immunization may also be beneficial if it could induce long-lasting immunity sufficient to reduce GBS colonization in the mother during pregnancy and/or adequate transfer of antibody to the infant to prevent both EOD and LOD. 

Another important issue in planning vaccination strategies is to consider prematurity and its impact on optimal timing of immunization, especially since GBS has been causally associated with preterm birth. No trials have specifically targeted preterm pregnancies yet to shed some light on this subject. Preterm infants may not benefit from immunization in late pregnancy because birth may occur before vaccine administration; thus, the protection against GBS disease will not be fully obtained. On the other hand, if multiple doses are required to optimize protection, early immunization in pregnancy would support completion of the vaccine series to benefit both term and preterm infants. However, immunization in early pregnancy may raise the concern about antibody waning by the time of delivery [72]. 

Moreover, delineating the best timing of immunization for both term and preterm infants requires sufficient data about the influence of timing on vaccine safety as well as the relevant immune responses, which is challenging in the absence of a correlate of protection. Safety and immunological data relating to the timing of maternal vaccination are still lacking. Most clinical trials have recruited women between 24 and 36 weeks of gestation [56,58,59,60,61,62]. Some on-going trials have lowered the gestational age to 21 weeks as part of their eligibility criteria [67,73]. Therefore, well-designed longitudinal studies are required to help inform the optimal GBS immunization schedule for pregnant people.

More trials are also required to investigate the durability of antibodies in the first 3 months of life for the optimal benefit of the infant. The transfer rate of transplacental antibodies and antibody waning by the time of delivery are influential factors in designing GBS vaccines against EOD and LOD. Additionally, multiple doses at different timings may be required to prevent EOD and LOD. While there are overlaps of serotypes causing EOD and LOD, an alternative strategy could be a GBS conjugate vaccine that includes most serotypes responsible for both EOD and LOD similar to approaches used in designing 20-valent pneumococcal conjugate vaccines with protection against 70% of the pneumococcal capsular serotypes causing disease [74].

Moreover, the conjugate vaccines that are currently under investigation do not include all GBS serotypes; thus, capsular switching or capsular replacement is a potential risk leading to the emergence of diseases caused by non-vaccine strains [3,75]. This phenomenon has also been described after pneumococcal conjugate vaccination [76].

Another concern regarding vaccination in pregnancy is the potential harm to the developing fetus. Using different databases, accumulated data from completed and ongoing studies on GBS vaccination in both low- and high-income countries that assessed pregnant women at different age groups (18–40) and gestational ages (21–35 weeks) showed no specific pattern of adverse outcomes for mothers or children (see Table 3). Like other non-live and inactivated vaccines, GBS vaccination is not expected to result in harm to the fetus, given the lack of safety concerns after vaccination with tetanus, diphtheria, pertussis, influenza [77], and COVID-19 vaccines [78]. Based on the findings of a systematic review, the Global Advisory Committee on Vaccine Safety also announced no safety concerns after immunization in pregnancy with inactivated vaccines in pregnancy [21,79].

Co-morbidities including HIV and malaria can also be problematic as they may cause insufficient placental antibody transfer [21]. This suggests that different protective antibody thresholds should be established in different settings when developing GBS vaccines. This is notably important in low-income countries such as sub-Sahara Africa where greater burden of GBS disease [80,81] and high prevalence of HIV in the pregnant population (up to 40%) have been reported [81,82,83]. 

The immunological interactions with other maternal and potentially infant vaccines should also be considered when scheduling for routine GBS immunization in prenatal care. There is a potential concern about the impact of carrier proteins of GBS conjugate vaccines on immune response via interfering with components of other vaccines including *Haemophilus influenzae* type b (Hib), meningococcal, and pneumococcal conjugate vaccines [84].

Finally, the global expansion of vaccine clinical trials into low-middle income countries (LMICs) with high burden of GBS disease is challenging [85]. Selecting trial participants from LMICs will benefit those communities by contributing to scientific advancements, capacity building of local medical infrastructure, and hopefully improving timely access to vaccines [86]. However, some LMICs may struggle with meeting international clinical science, regulatory, and ethical standards as they still need to build adequate local scientific capacity and strengthen areas such as good clinical practice (GCP), good laboratory practice (GLP), laboratory infrastructure and training, data management, and epidemiology [86,87]. Some of the issues encountered in LMICs settings include lack of procedures for reviewing study protocols and obtaining culturally sensitive informed consent, lack of resources leading to delayed timelines, and lack of monitoring systems and standard of care that should be used in research [86]. However, to ensure that collected safety and efficacy data from clinical trials are generalizable to broader populations, clinical trials should consider the potential differences in environmental factors across regions such as differences in local medical practices and prevalence of comorbidities such as HIV which is more common in LMICs where the vaccine is targeted for use [86]. Accordingly, it is an obligation of the global scientific community to ensure development of GBS vaccines that can be deployed worldwide is supported, and current challenges in LMICs should be addressed by strengthening infrastructure and developing capacity to support the increasing conduct of vaccine clinical trials, supported by both LMICs and high-income countries (HICs) [86]. 

## 12. Conclusions 

In summary, no GBS vaccine is approved yet but trials evaluating immunization of pregnant women against GBS hold promising results for the reduction of neonatal GBS disease. While both polysaccharide–protein conjugate and protein subunit vaccines have successfully reached phase II clinical trials, the hexavalent conjugate vaccine seems to be the best potential candidate as it covers most invasive serotypes. The immunogenicity and safety of the hexavalent conjugate vaccine among non-pregnant women are also optimal; however, the data from the pregnant population are needed to evaluate its use in maternal immunization. Clinical trials should be prioritized in pregnant people for any new vaccine candidates. GBS vaccination in the second or early third trimester may be the ideal timing for best protection of the newborn and infant. However, along with the safety, comprehensive knowledge of the immune response and its duration are required to determine the best timing to benefit both term and preterm babies. The next step could include clinical phase III efficacy trials and considering how to obtain high vaccine coverage as a requirement to reduce global burden of neonatal GBS disease.

## Figures and Tables

**Table 2:** Summary of probiotics trials in pregnant populations against GBS colonization.

Study (NCT Registration)	Country/ Region	Study Design	Study Population, N	Gestational Age (Weeks)	Mean Age in Years (SD)	Therapy	Treatment Course	Efficacy		Preterm Birth	
								n/total (%)	*p* value	n/total (%)	*p* value
Farr et al., 2020 (NCT03008421) [32]	Austria	Randomized clinical trial	82	33–37	Probiotic:38.5 ± 1.4; Placebo: 38.6 ± 1.2	A dietary probiotic supplement (containing *L. jensenii, L. crispatus, L. rhamnosus,* and *L.gasseri* ) or placebo	Twice daily	GBS positivity: Probiotic = 12/33 (63.6%); Placebo = 6/27 (77.8%)	0.24	Probiotic = 4 (9.8%); Placebo = 1 (2.4%)	0.20
Olsen et al., 2018 [34]	Australia	Randomized clinical trial	34	36	Probiotic:32 ± 4; Standard: 30 ± 4.3	A daily oral dose of probiotics (containing *L. rhamnosus and L. fermentum/reuteir)*; Placebo arm: Standard antenatal care	Once daily for 3 weeks	GBS negativity: Probiotic = 4/19 (21.1%); Standard of Care = 3/21 23.1%)	0.7	N/A	
Hanson et al., 2014 [31]	US	Open label, two group quasi experiment	20	28, 32, 36	Probiotic:25.8 ± 3.8; Control:25.9 ± 5.1	oral probiotic (containing *L. acidophillus, B. lactis*, and *B. longum*) or placebo	Once daily	GBS positivity: Probiotic= 2/10 (20%); Standard of Care = 3/10 (20%)	>0.05	N/A	
Sharpe et al., 2021 [35]	Canada	Double blind randomized control pilot trial	139	35–37	Probiotic:32.04; Placebo:32.47	Two capsules of probiotics (*L. rhamnosus and L. reuteri*) or placebo	Twice daily	GBS positivity: Probiotic = 9/57(15.8%); Placebo = 12/56 (21.4%)	0.48	N/A	
Ho et al., 2016 (NCT03688321) [33]	Taiwan	Prospective, double-blind randomized clinical trial	110	35–37	Probiotic:32.0 ± 4.0; Placebo:32.0 ± 3.7	Two probiotic capsules (containing *L. rhamnosus and L. reuteri*) before bedtime or placebo	Once daily	GBS negativity: Probiotic = 21/49 (42.9%); Placebo = 9/50 (18%)	0.007	N/A	
Aziz 2017 (NCT01479478) [36]	US	Randomized clinical trial	251	35–37	Probiotic:31.4 ± 5.7; Placebo; 31.9 ± 5.3	Probiotic dietary supplement (containing *L. rhamnosus and L. reuteri*) or placebo	Once daily	GBS positivity: Probiotic= 20/108 (18.5%); Placebo= 23/117 (19.7%)	0.87	N\A	

## Data Availability

Not applicable.

## Supplementary Materials

The following supporting information can be downloaded at: https://www.mdpi.com/article/10.3390/pathogens12020229/s1, Table S1: Detailed adverse outcomes of GBS Vaccines investigated in clinical trials on pregnant or non-pregnant populations using different databases: Table S2: Additional reports on GBS Vaccines investigated in clinical trials on pregnant or non-pregnant populations using different databases.
