# Supplementary material for: Strategies to Prevent Early and Late-Onset Group B Streptococcal Infection via Interventions in Pregnancy
Source: Pathogens. 2023 Feb 1;12(2):229. doi: 10.3390/pathogens12020229 (PMC9959229; doi:10.3390/pathogens12020229)
Supplement: Supplementary file 1 [file pathogens-12-00229-s001.zip › pathogens-2088022 -SM.pdf]

Supplemental Table S1: Detailed adverse outcomes of GBS Vaccines investigated in clinical trials on pregnant or non-pregnant populations using different databases

Vaccine Formulation	Registry Number	Country	Clinical Phase (Status)	Study Duration	Age group, years	Gestation Weeks	Total Study Population	Population, N (Groups)	Local reactions	Systemic events	Reference
**Monovalent GBS III-TT conjugate vaccine**	N.R.	Canada	N.R.	NR	18-40	Non-pregnant	100	Vaccine group:<br>III-TT58 mg: 30<br>III-TT 14.5 mg: 15<br>III-TT 3.6 mg: 15<br>III CPS, 50 mg: 30<br>Placebo group:<br>Saline, NA: 10	Vaccine group:<br>III-TT,58µg group:<br>Pain- sore with movement: 9/30 (30%)<br>Redness and swelling- sore with movement: 3/30(10%)<br>14.5µg group:<br>Pain- sore with movement: 5/15 (33%)<br>3.6µg group:<br>Pain- sore with movement: 1/15 (7%)<br>III CPS, 50µg group:<br>Pain- sore with movement: 4/30 (13%)<br>Redness and swelling- sore with movement: 1/30 (3%)<br>Placebo group:<br>Saline, N.A group:<br>Pain- sore with movement: 3/10 (30%)	N.R.	Kasper et al, 1996 [53]
**Monovalent GBS III-TT conjugate vaccine**	N.R.	USA	Phase 1 (Completed)	NR	18-45	>37	30	Vaccine group: 20<br>Placebo group: 10	Vaccine group:<br>Injection site pain-<br>mild: 11/18 (65%)<br>moderate: 1/18 (5%)<br>Redness at the injection site: 2/18 (10%)<br>Placebo group:<br>Injection site pain, mild or moderate: 5/12 (40%)<br>Redness at the injection site: 0/12 (0%)	N.R.	Baker et al, 2003a [54]
**Bivalent GBS conjugate**	N.R.	USA	Phase 2 (Completed)	NR	18-45	Non-pregnant	75	Vaccine group:<br>GBS II–TT (3.6 mg of CPS): 25<br>GBS III–TT (12.5 mg of CPS): 25<br>GBS II–TT/III–TT vaccine: 25	Monovalent GBS-II-TT//IIITT Group:<br>Mild redness or swelling at the injection site: 2/ 25(8%)<br>Bivalent GBS-II-TT/III-TT Group:<br>Mild redness or swelling at the injection site: 2/ 25(8%)	Monovalent GBS-II-TT//IIITT Group: No serious adverse effects were observed.<br>Mild systemic symptoms associated with low-grade fever: 2/75 (2.6%) in males<br>Bivalent GBS-II-TT/III-TT Group:	Baker et al, 2003b [56]

								Placebo group:<br>No placebo group		Single temperature elevation to 100.4F 17 h after receiving the bivalent vaccine 1/75 (1.3%) in males	
**Trivalent GBS conjugate vaccine**	NCT01193920	South Africa	Phase1b/2 (Completed)	2010-2011	18-40	28-35	380	Vaccine group (Non-pregnant):<br>GBS vaccine 20 µg: 40<br>Placebo group: (Non-pregnant):<br>Placebo: 20<br>Vaccine group (Pregnant)<br>GBS vaccine 0·5 mg : 80<br>GBS vaccine 2·5 µg : 80<br>GBS vaccine 5·0 µg: 80<br>Placebo Group (Pregnant)<br>Placebo: 80	Vaccine group (Non-pregnant):<br>GBS vaccine 20 µg group:<br>Injection site pain: 39/40 (97.50%)<br>Swelling: 14/40 (35.00%)<br>Placebo group: (Non-pregnant):<br>Injection site pain:15/20 (75.00%)<br>Swelling: 2/20 (10.00%)<br>Vaccine group (Pregnant)<br>GBS vaccine 0·5 mg group<br>Injection site pain: 28/80 (35.00%)<br>Swelling: 2/80 (2.50%)<br>GBS vaccine 2·5 µg group<br>Injection site pain: 25/80 (31.25%)<br>Swelling: 5/80 (6.25%)<br>GBS vaccine 5·0 µg group<br>Injection site pain: 26/80 (32.50%)<br>Swelling: 5/80 (6.25%)<br>Placebo Group (Pregnant) group<br>Injection site pain: 24/80 (30.00%)<br>Swelling: 1/80 (1.25%)	Vaccine group (Non-pregnant):<br>GBS vaccine 20 µg group<br>Chills: 15/40 (37.50%)<br>Fatigue: 29/40 (72.50%)<br>Pyrexia: 6/40 (15.00%)<br>Placebo group: (Non-pregnant):<br>Chills: 6/20 (30.00%)<br>Fatigue: 12/20 (60.00%)<br>Pyrexia: 0/20 (0.00%)<br>Vaccine group (Pregnant)<br>GBS vaccine 0·5 mg group:<br>Chills: 16/80 (20.00%)<br>Fatigue: 39/80 (48.75%)<br>Pyrexia: 5/80 (6.25%)<br>GBS vaccine 2·5 µg group<br>Chills: 7/80 (8.75%)<br>Fatigue: 34/80 (42.50%)<br>Pyrexia: 1/80 (1.25%)<br>Death: 1/80 (1.25%)<br>GBS vaccine 5·0 µg group<br>Chills: 8/80 (10.00%)<br>Fatigue:43/80 (53.75%)<br>Pyrexia: 4/80 (5.00%)<br>Placebo Group (Pregnant)<br>Chills: 9/80 (11.25%)<br>Fatigue:37/80 (46.25%)<br>Pyrexia: 2/80 (2.50%)	Madhi et al, 2016 [58]
**Trivalent GBS conjugate vaccine**	NCT01412801	Malawi, South Africa	Phase 2 (Completed)	2011-2012	18-40	24-35	270	GBS vaccine in:<br>HIV-infected, low CD4 cell count group: 91<br>HIV-infected, high CD4 cell count	GBS vaccine in:<br>HIV-infected, low CD4 cell count >50 to ≤350 cells per µl- Pain and swelling at an injection site:16/87 (18%)<br>HIV-infected, high CD4 cell count >350 cells per µL- Pain and swelling at an	GBS vaccine in:<br>HIV-infected, low CD4 cell count >50 to ≤350 cells per µL- any systemic reactions (chills/ nausea/ malaise/ myalgia/ arthralgia/	Heyderman et al, 2016 [59]

								group: 89<br>HIV-uninfected group: 90	injection site: 26/88 (30%)<br>HIV-uninfected- Pain and swelling at an injection site: 35/90 (39%)	headache/ fatigue/ rash/ fever) : 35/87 (40%)<br>HIV-infected, high CD4 cell count >350 cells per μL- any systemic reactions (chills/ nausea/ malaise/ myalgia/ arthralgia/ headache/ fatigue/ rash/ fever) : 48/88 (55%)<br>HIV-uninfected- any systemic reactions (chills/ nausea/ malaise/ myalgia/ arthralgia/ headache/ fatigue/ rash/ fever) : 53/90 (59%)	
**Trivalent GBS conjugate vaccine**	NCT01446289/ EUCTR2010-020840-36-BE	Belgium, Canada, Italy	Phase 2 (Completed)	2011-2013	18-40	24-35	86	Vaccine group: 49<br>Placebo group: 34	Vaccine group:<br>Pain: 35/49 (71%)<br>Swelling: 4/49 (8.1%)<br>Ecchymosis: 2/49 (4%)<br>Erythema: 6/49 (12%)<br>Placebo group:<br>Pain: 12 /34 (35%)<br>Swelling: 0/34 (0%)<br>Ecchymosis: 0/34 (0%)<br>Erythema: 0/34 (0%)	Vaccine group:<br>Chills: 4/49 (8%)<br>Malaise:14/49 (28%)<br>Myalgia:27/49 (55%)<br>Arthralgia: 4/49 (8%)<br>Headache: 16/49 (32%)<br>Fatigue: 31/49 (63%)<br>Placebo group:<br>Chills: 3/34 (8%)<br>Malaise: 9/34 (26%)<br>Myalgia: 3/34 (8%)<br>Arthralgia: 9/34 (26%)<br>Headache: 21/34 (61%)<br>Fatigue: 26/34 (76%)	Donders et al, 2016 [60]
**Trivalent GBS conjugate vaccine**	NCT02046148	USA	Phase 2 (Completed)	2014-2015	18-40	24-35	75	Vaccine group: 49<br>Placebo group: 26	Vaccine group:<br>Pain at injection site including redness: 24/49 (50%)<br>Placebo group:<br>Pain at injection site including redness: 8/26 (31%)	Vaccine group:<br>Fatigue: 18/49 (38%)<br>Nausea: 7/49 (15%)<br>Headache: 6/49 (13%)<br>Loss of apetite:6/49 (13%)<br>Myalgia: 2/49 (4%)<br>Arthralgia: 2/49 (4%)<br>Chills: 1/49 (2%)<br>Rash: 1/49 (2%)<br>Urticaria: 1/49 (2%)<br>Placebo group:	Swamy et al, 2020[62]

										Fatigue: 6/26 (23%)<br>Nausea: 3/26 (12%)<br>Headache: 3/26 (12%)<br>Loss of apetite:2/26 (8%)<br>Myalgia: 2/26 (8%)<br>Arthralgia: 2526 (8%)<br>Chills: 1/26 (4%)	
**Quadrivalent GBS**	N.R.	USA	Phase 1 (Completed)	NR	18-40	Non-pregnant	40	Vaccine group: 40<br>Placebo group: Absence of placebo group	Vaccine group: Pain, tenderness, and erythema: 6/40 (15%)<br>Placebo group: No placebo group	Vaccine group: nausea, malaise, myalgia, or headache: 6 (15%)<br>Placebo group: No placebo group	Kotloff et al,1996 [64]
**Hexavalent GBS conjugate vaccine**	NCT03170609	USA	Phase 1/2 (Completed)	2017-2018	18-49	Non-pregnant	365	Vaccine group:<br>GBS6 5 μg with AlPO4: 52<br>GBS6 5 μg without AlPO4: 52<br>GBS6 10 μg with AlPO4: 52<br>GBS6 10 μg without AlPO4: 52<br>GBS6 20 μg with AlPO4: 52<br>GBS6 20 μg without AlPO4: 52<br>Placebo group: 52	Vaccine group: GBS6 5 μg without AlPO4 group: pain at the injection site: 13/52 (25%)<br>GBS6 20 μg with AlPO4 group: Pain at an injection site: 28 (54%)<br>Placebo group: No severe local reactions were reported	Vaccine group: fatigue or tiredness and headache: 1/52(2%)<br>Placebo group: fatigue or tiredness and headache: 1/52(2%)	Absalon et al, 2021 [65]

NR: Not reported

Supplemental Table S2: Additional reports on GBS Vaccines investigated in clinical trials on pregnant or non-pregnant populations using different databases

Vaccine Formulation	Registry Number	Country	Clinical Phase (Status)	Study Duration	Age group, years	Gestation Weeks	Total Study Population	Population, N (Groups)	Time point for immunogenicity assessment	Immunogenicity results: Mean GMC (95% CI)	Reference
**Trivalent GBS conjugate vaccine**	NCT01193920	South Africa	Phase 1b/2 (Completed)	2010-2012	0	N.A	317	Vaccine group (After birth): GBS serotype Ia GBS 0.5 mg: 75 GBS 2.5 mg: 76 GBS 5.0 mg: 69 GBS serotype Ib GBS 0.5 mg: 71 GBS 2.5 mg: 71 GBS 5.0 mg: 66 Placebo group (After birth): GBS serotype Ia Placebo: 73 GBS serotype Ib Placebo: 62	At delivery	Infants: Vaccine group: GBS serotype Ia: 6.66 (4.03–11.0) serotype Ib: 1.15 (.68–1.95) serotype III: 2.11 (1.26–3.54) Placebo group GBS serotype Ia: 0.49 (.34–.71) serotype Ib: 0.21 (.14–.32) serotype III: 0.29 (.19–.43)	Madhi et al, 2017 [57]
**Trivalent GBS conjugate vaccine**	NCT01446289	Italy, Belgium, Canada, USA	Phase 2 (Completed)	2011-2013	18-40	24-35	86	Vaccine group: 51 Placebo group: 35	At delivery	Vaccine group: Serotype Ia- no. of OPKA ≥30: 32 (97%) ELISA GMC (μg/mL): 3.3 Serotype Ib- no. of OPKA ≥30: 20 (61%) ELISA GMC (μg/mL): 3.6 Serotype III- no. of OPKA ≥30: 29 (88%) ELISA GMC (μg/mL): 2.9 Placebo group: Serotype Ia- no. of OPKA ≥30: 12 (55%) ELISA GMC (μg/mL): 0.5 Serotype Ib- no. of OPKA ≥30: 3 (14%) ELISA GMC (μg/mL): 0.1 Serotype III- no. of OPKA ≥30: 9 (41%) ELISA GMC (μg/mL): 0.1	Fabrini et al, 2018 [61]

Footnote:

Clinical trial registries maintained by WHO, US National Library of Medicine's Clinical Trial database, EU Clinical Trials Register, Health Canada's Clinical trial search database, Chinese Clinical Trial Registry, BMC's ISRCTN registry, Clinical Trial Registry-India for GBS vaccine trials in pregnant women studying prevention of GBS infections in their infants.

GBS: *Group B Streptococcus*; SAE: Serious adverse events, AE: Adverse events, CPS: capsular polysaccharide, HIV: Human immunodeficiency virus, N.R: not reported, OPKA: opsonophagocytic bacterial killing assay, ELISA: The enzyme-linked immunosorbent assay, GMC: geometric mean concentration, sIgA: serotype-specific secretory immunoglobulin A, IgG: immunoglobulin G.
